# Immune Checkpoint Function of CD85j in CD8 T Cell Differentiation and Aging

**DOI:** 10.3389/fimmu.2017.00692

**Published:** 2017-06-14

**Authors:** Claire E. Gustafson, Qian Qi, Jessica Hutter-Saunders, Sheena Gupta, Rohit Jadhav, Evan Newell, Holden Maecker, Cornelia M. Weyand, Jörg J. Goronzy

**Affiliations:** ^1^Division of Immunology and Rheumatology, Department of Medicine, Stanford University, Stanford, CA, United States; ^2^Department of Medicine, Palo Alto Veterans Administration Healthcare System, Palo Alto, CA, United States; ^3^Department of Microbiology and Immunology, Stanford University, Stanford, CA, United States; ^4^Agency for Science, Technology and Research (A*STAR), Singapore Immunology Network (SIgN), Singapore, Singapore

**Keywords:** ILT-2, LILRB1, immunosenescence, exhaustion, NK receptors, innate-like CD8 T cells, cytomegalovirus, chronic viral infection

## Abstract

Aging is associated with an increased susceptibility to infection and a failure to control latent viruses thought to be driven, at least in part, by alterations in CD8 T cell function. The aging T cell repertoire is characterized by an accumulation of effector CD8 T cells, many of which express the negative regulatory receptor CD85j. To define the biological significance of CD85j expression on CD8 T cells and to address the question whether presence of CD85j in older individuals is beneficial or detrimental for immune function, we examined the specific attributes of CD8 T cells expressing CD85j as well as the functional role of CD85j in antigen-specific CD8 T cell responses during immune aging. Here, we show that CD85j is mainly expressed by terminally differentiated effector (TEMRAs) CD8 T cells, which increase with age, in cytomegalovirus (CMV) infection and in males. CD85j^+^ CMV-specific cells demonstrate clonal expansion. However, TCR diversity is similar between CD85j^+^ and CD85j^−^ compartments, suggesting that CD85j does not directly impact the repertoire of antigen-specific cells. Further phenotypic and functional analyses revealed that CD85j identifies a specific subset of CMV-responsive CD8 T cells that coexpress a marker of senescence (CD57) but retain polyfunctional cytokine production and expression of cytotoxic mediators. Blocking CD85j binding enhanced proliferation of CMV-specific CD8 T cells upon antigen stimulation but did not alter polyfunctional cytokine production. Taken together, these data demonstrate that CD85j characterizes a population of “senescent,” but not exhausted antigen-specific effector CD8 T cells and indicates that CD85j is an important checkpoint regulator controlling expansion of virus-specific T cells during aging. Inhibition of CD85j activity may be a mechanism to promote stronger CD8 T cell effector responses during immune aging.

## Introduction

Human aging is characterized by a loss of effective immune responses against viral pathogens, coinciding with an increased susceptibility to infection and a failure to control latent viruses (1, 2). Functional CD8 T cell responses are critical for protection against viral infections. However, there are considerable changes in the memory CD8 population that may contribute to reduced functionality during immune aging. These changes include an accumulation of terminal differentiated effector CD8 T cells, frequently labeled as “senescent,” that exhibit reduced proliferative capacity but maintain cytotoxic and cytokine-producing functions, unlike exhausted T cells that lack both proliferative and effector responses (3, 4). Acquisition of such CD8 T cells is also a hallmark of chronic viral infections, such as cytomegalovirus (CMV), and further accelerated in CMV antibody-positive older individuals (5, 6). Moreover, recent evidence suggests that end-differentiated CD8 T cells demonstrate similar properties to innate-like T cells and gain the expression of multiple activating and inhibiting regulatory receptors including killer immunoglobulin-like receptors (7, 8).

The increased expression of the inhibitory receptor CD85j (alternatively known as ILT-2 or LILRB1) on CD8 T cells is one phenotypic hallmark of aging (9, 10). A wide range of immune cells including monocytes, B cells, dendritic cells, and a subset of NK and T cells express CD85j. However, the levels of CD85j on cell types vary widely based on cell-specific transcriptional regulation of CD85j (11). CD85j shares structural similarities with PD-1, a well-established checkpoint molecule, which include a cytoplasmic tail containing multiple immunoreceptor tyrosine-based inhibitory motifs (ITIMs) that are able to recruit tyrosine phosphatases SHP-1 and SHP-2 and a tyrosine-based switch motif (ITSM) (12–14). CD85j recognizes a broad range of classical and non-classical MHC class I molecules, including high affinity for HLA-G (15–17). CD85j also binds with high affinity to a CMV MHC class I homolog UL18 (18, 19), is expressed by CMV-specific CD8 T cells (20), and is proposed to play a significant role in CD8 T cell responsiveness to CMV infection. Thus, CD85j may have an important function in checkpoint inhibition, maintenance of T cell homeostasis, and prevention of memory inflation with aging and CMV infection.

Previous studies suggest that CD85j can inhibit cytokine production, decrease proliferation, and reduce cytotoxicity of T cells (21, 22), which are common features of exhausted CD8 T cells. However, CD85j is often coexpressed with the senescence marker CD57 (23). Thus, it is unclear whether CD85j-expressing CD8 T cells in immune aging are truly exhausted or a subset of senescent cells. Moreover, the specific function of CD85j on CD8 T cells during chronic viral infection in older individuals is unknown. Thus, to better define the biological significance of CD85j expression on CD8 T cells and to address the question whether inhibition of CD85j in older individuals is beneficial or detrimental for immune function, we examined the specific attributes of CD8 T cells expressing CD85j as well as the functional role of CD85j in CMV-specific CD8 T cell responses during immune aging.

## Materials and Methods

### Study Participants

This study included samples from three sources. We obtained data and additional peripheral blood samples from a previously published cohort of 740 healthy individuals aged 40–97 that approximately mirrored San Francisco Bay Area ethnic demographics (24). Additional healthy individuals between the age of 18 and 80 years from the same draw area were recruited. Deidentified samples from HLA-A*02^+^ individuals of various ages and with positive CMV serology were purchased from the Stanford Blood Center (Palo Alto, CA, USA). The study was in accordance with the Declaration of Helsinki, approved by the Stanford Institutional Review Board, and all participants gave written informed consent.

### Cellular Phenotyping by Flow Cytometry

For cellular phenotyping of tetramer-specific cells, we used CD3-APC/Cy7, CD8-qDot605, CD45RA-PE/Cy7, CCR7-PerCp/Cy5.5, CD28-PE, CD85j (ILT-2)-APC, and tetramer–Pacific Blue. Antibodies were purchased from BD Bioscience, Biolegend, or eBioscience. HLA-A*0201 monomers loaded with peptides for CMV pp65 (NLVPMVATV) or EBV BRLF1 (YVLDHLIVV) were tetramerized and labeled with streptavidin–Pacific Blue.

### T Cell Sorting and *TRB* Sequencing

Total T cells were isolated by negative selection using human T cell RosetteSep enrichment kit (StemCell Technologies) from platelet donor apheresis lymphocytes of HLA-A2 donors who are CMV seropositive. T cells were stained with CD4, CD8, pp65 HLA-A*0201 tetramer, and CD85j antibodies. CD85j^+^ and CD85j^−^ pp65-HLA-A*0201 tetramer^+^ CD8 T cells were sorted using a FACSAria (BD Bioscience) and split into two to four replicates with 4,000–5,000 cells per replicate. Total RNA was extracted from each T cell replicate using RNeasy Plus Micro kit (Qiagen), followed by generation of cDNA using SuperScript VILO master mix (Invitrogen). The amplification and sequencing of TRB gene libraries followed the protocol as previously described (25).

The sequences were mapped to human *TRB* reference sequences as described in detail previously (25, 26). Clonotypes were defined as sequences with the same *TRBV* and *TRBJ* gene segments and identical CDR3 amino acid sequences. In addition, any clonotype that was only found in one replicate library was filtered out of the analysis. The clonality index for each population was calculated using the lymphclon package (https://arxiv.org/abs/1408.1149) (25).

### CyTOF

PBMCs were left unstimulated or stimulated for 18 h with CMV peptide pools in the presence of brefeldin A and monensin (BD Bioscience). For CMV-specific stimulation, two peptide super pools, each made up of overlapping peptide pools of four different antigens, were used. The immediate early (IE) pool consisted of IE-1, IE-2, US3, and UL36. The late pool consisted of pp65, UL32, UL48AB, and UL55 (gB), based on previously described work (27). Following stimulation, cells were resuspended in CyFACS buffer (1× PBS with 0.1% BSA, 2 mM EDTA, and 0.5% sodium azide) and stained with isotope-tagged antibodies before being acquired on the CyTOF. For a detailed protocol, see http://iti.stanford.edu/himc/protocols.html (CyTOF ICS protocol) and Table S1 in Supplementary Material.

Data acquired from CyTOF were initially analyzed using FlowJo v10.1 (FlowJo Inc.). CD3^+^CD19^−^CD8^+^CD4^−^ cells expressing CD107a or one of the following cytokines, IFNγ, TNFα, IL-2, GM-CSF, or MIP1β, after stimulation with IE or late pool were considered “CMV-responsive” CD8 T cells. The CMV-responsive cells for 30 individuals were concatenated, and cluster analysis was performed using X-shift (28). For final clustering, basic phenotypic (CD45RA, CCR7, CD28, CD27, and CD127) and the six preselected response factors were excluded.

### *In Vitro* Blocking Experiments

#### Reagents

Peptide pools were purchased from JPT Peptide Technologies. The late antigen pp65 peptide pool was a combination of 138 peptides derived from a peptide scan (15mers with 11 amino-acid overlap) through 65 kDa phosphoprotein (pp65) (Swiss-Prot ID: P06725) of human cytomegalovirus (HHV-5). The immediate early antigen IE-1 peptide pool was a combination of 120 peptides derived from a peptide scan (15mers with 11 amino acid overlap) through 55 kDa immediate early protein 1 (IE-1) (Swiss-Prot ID: P13202) of human cytomegalovirus (HHV-5).

#### PBMC Assays

PBMCs were stimulated with pp65 or IE-1 peptide pools in the presence of brefeldin A. Monoclonal IgG_2B_ mouse anti-human CD85j (ILT-2) antibody (R&D Systems) or an isotype control (eBioscience) (5 μg/mL) was added prior to stimulation. For cytokine production, cells were stimulated for 13 h. For proliferation, cells were prelabeled with CFSE and stimulated for 7 days. Following stimulation, cells were resuspended in FACS buffer and stained with fluorescently tagged antibodies before being acquired on the flow cytometer. The following anti-human antibodies were used: CD3-APC/Cy7, CD8-qDot605, CD85j-APC, IFNγ-PE/Cy7, and TNFα-AF700.

#### Tetramer-Induced T Cell Proliferation Assay

Total T cells were isolated from PBMCs using untouched human T cells Dynabead kit (Fisher Scientific) and prelabeled with CFSE. Cells (1 × 10^6^ per mL) were added to 96-well plates precoated with CMV_pp65_ or HIV_gag_ (SLYNTVATL) peptide-loaded HLA-A*0201 monomers (400 ng/mL) in the presence of soluble anti-CD85j or isotype control antibody (5 μg/mL). Following 7 days of stimulation, cells were resuspended in FACS buffer and stained with fluorescently tagged antibodies (same as baseline phenotyping) before being acquired on the flow cytometer.

### Statistics

Statistical analyses, including Spearman correlation coefficients and non-parametric testing, were performed using GraphPad Prism v6 (GraphPad, San Diego, CA, USA). All *p*-values were derived using two-tailed tests and *p* < 0.05 were considered significant.

## Results

### Correlates of CD85j Expression on CD8 T Cells in a Healthy Population

**Figure 1:**
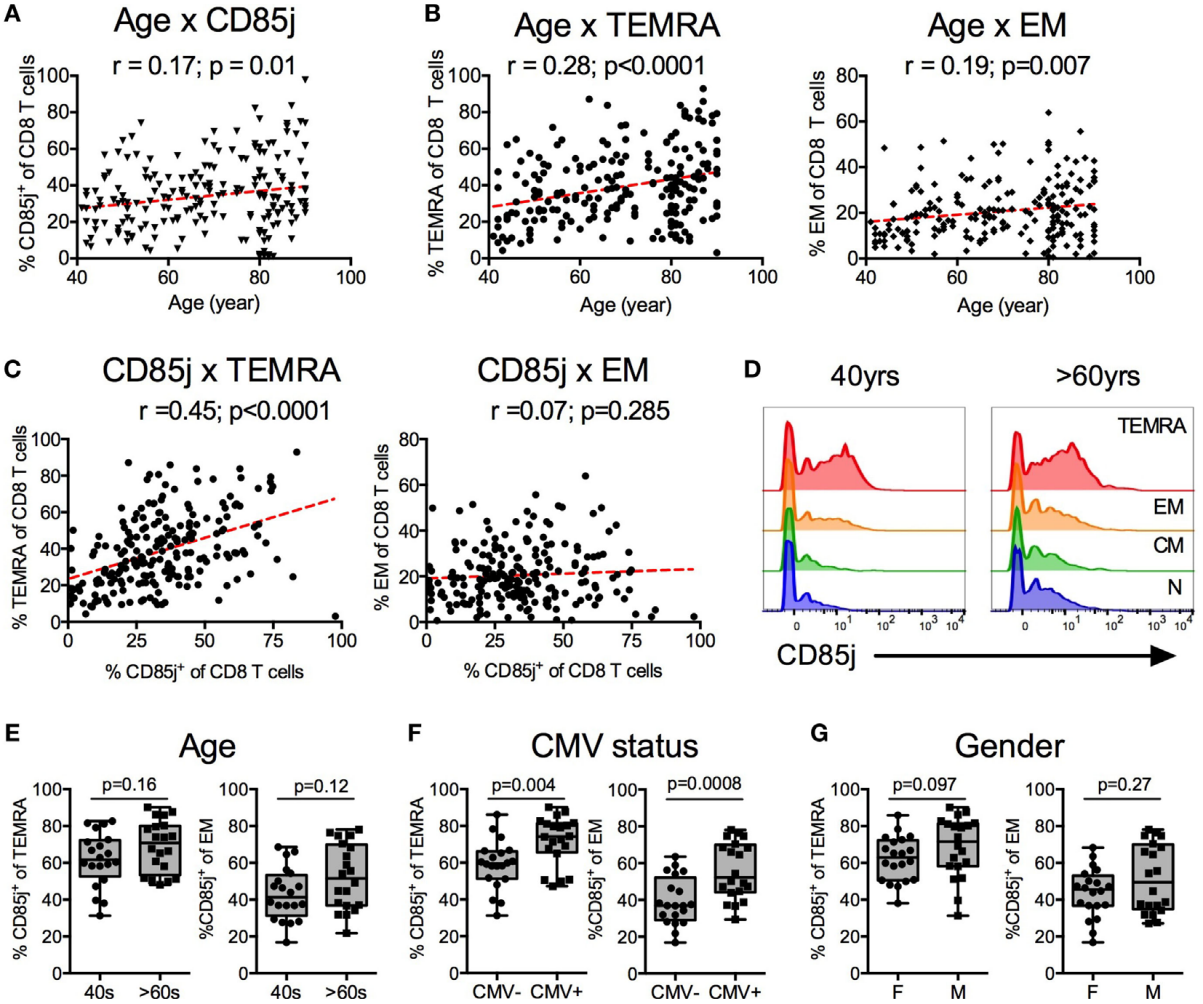
CD85j expression on CD8 T cells and T cell subsets by age, gender, and cytomegalovirus (CMV) status. Spearman correlations between age and the percentage of **(A)** CD85j^+^ CD8 T cells, **(B)** TEMRA (CD45RA^+^CCR7^−^) CD8 T cells (left), and effector memory (EM; CD45RA^−^CCR7^−^) CD8 T cells (right) from 210 healthy individuals. **(C)** Spearman correlations between percentage of CD85j^+^ CD8 T cells and TEMRA (left) or EM (right) subsets. **(D)** Representative histograms of CD85j expression on naïve (CD45RA^+^CCR7^+^), CM (CD45RA^−^CCR7^+^), EM, and TEMRA CD8 T cell subsets in mid-age (40 years) and older (>60 years) adults. **(E–G)** Frequency of CD85j expression on TEMRA and EM CD8 T cell subsets by **(E)** age [mid-age (40 s) and older (>60 s) adults], **(F)** CMV status [CMV-negative (CMV^−^) and CMV-positive (CMV^+^) individuals], or **(G)** gender [females (F) and males (M)]. *p*-values in **(E–G)** were determined using the Mann–Whitney test.

We first characterized the relationship of CD85j^+^ cells to age and to specific CD8 T cell subsets in a cohort of 210 healthy individuals that has been previously described (24). CD85j^+^ CD8 T cells increased with age (Figure 1A). Similarly, terminal differentiated effector CD8 T cells (termed “TEMRA”) increased with age, with a slope and correlation coefficient similar to that of CD85j^+^ cells (Figure 1B). Effector memory (EM) and naïve (N) CD8 cell frequencies also correlated with age (EM: *p* = 0.007, *r* = 0.19; N: *p* < 0.0001, *r* = −0.5) (Figure 1B; Figure S1A in Supplementary Material). As TEMRA and EM populations both showed similar positive correlations with age as the CD85j^+^ population, we next asked which CD8 T cell subset, TEMRA or EM, most closely correlated with CD85j^+^ cell frequencies. CD85j^+^ cells positively correlated with TEMRAs (Figure 1C). No correlation between EM and CD85j^+^ cell frequencies was found. Furthermore, CD85j^+^ cells negatively correlated with naïve CD8 T cells (N: *p* < 0.0001, *r* = −0.405) (Figure S1B in Supplementary Material). We also observed increased frequencies of CD85j^+^ cells and TEMRA cells in CMV-positive individuals and in males (Figures S1C,D in Supplementary Material).

To confirm that CD85j is expressed on TEMRA and whether increased CD85j^+^ cells with age is a result of increased frequencies of TEMRAs with age, we analyzed CD85j expression on T cell subsets in a subcohort of 20 mid-age (40–50 years) and 20 older (>60 years) individuals. Indeed, CD85j is expressed by TEMRAs, with a small fraction of CD85j^+^ EM T cells (Figure 1D). Central memory and naïve cells had very few CD85j^+^ cells. Of note, CD85j-intermediate staining is almost exclusively observed within the effector T cell populations. Naïve and CM populations lack this population and the low CD85j staining observed in naïve and CM cells is seen in all populations of cells. With age, there was a trend for increased percentage of CD85j^+^ TEMRA and EM, however, the difference did not reach statistical significance in our small subcohort (Figure 1E). We also compared the frequencies of CD85j^+^ cells within T cell subsets by gender and CMV status within this subcohort. CMV-positive individuals had higher frequencies of CD85j^+^ TEMRAs and CD85j^+^ EM CD8 T cells compared with CMV-negative individuals (Figure 1F). Gender also has a modest effect of the frequencies of CD85j^+^ TEMRAs, with males exhibiting slightly higher frequencies than females (Figure 1G). Age only had a minor effect on CD85j expression by effector subsets, not reaching significance. On the other hand, CMV infection caused a pronounced increase in CD85j^+^ TEMRAs, regardless of age. Thus, mainly terminal differentiated effector CD8 T cells express CD85j and multiple factors including differentiation state, CMV infection, gender, and age influence the frequencies of CD85j^+^ T cells and effector T cell subsets.

### Expression of CD85j on CMV and EBV-Specific Effector CD8 T Cells

**Figure 2:**
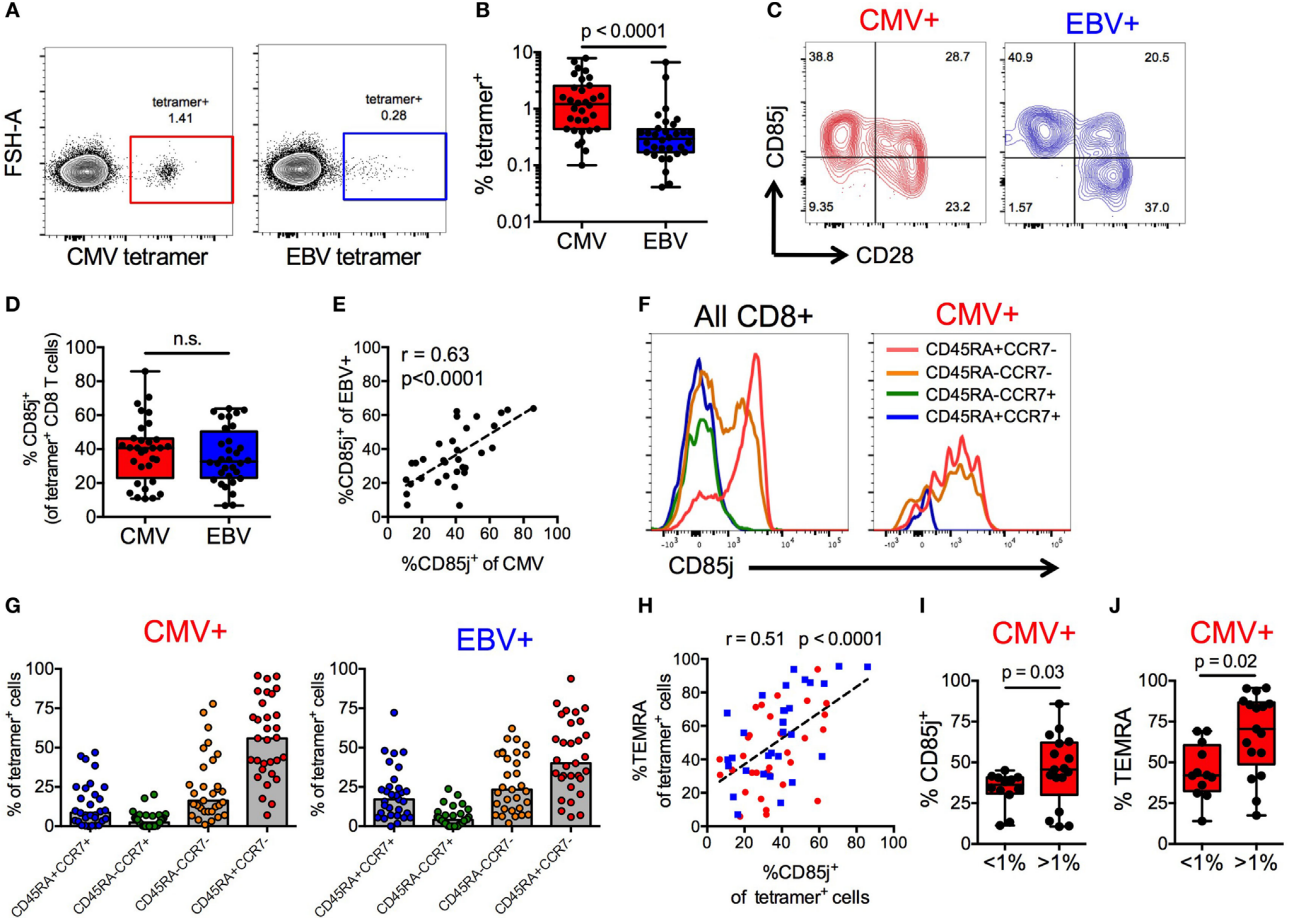
CD85j expression on cytomegalovirus (CMV)- and EBV-specific CD8 T cells. **(A)** Representative flow plot of CMV (pp65) and EBV (BRLF1) tetramer staining on CD8 T cells from older individuals. **(B)** Frequencies of CMV- and EBV-specific CD8 T cells in total T cells from CMV^+^ older individuals (*n* = 32). **(C)** Representative flow plot of CD85j and CD28 staining on CMV- and EBV-specific CD8 T cells. **(D)** Frequencies of CD85j^+^ cells in CMV- and EBV-specific CD8 T cells. **(E)** Spearman correlation between the frequencies of CD85j^+^ cells in CMV-specific and EBV-specific CD8 T cells. **(F)** Representative histograms of CD85j expression in gated subsets of total CD8^+^ T cells and of CMV-specific CD8 T cells. **(G)** The frequencies of T cell subsets in CMV- and EBV-specific CD8 T cells defined as naïve (CD45RA^+^CCR7^+^), central memory (CD45RA^−^CCR7^+^), effector memory (CD45RA^−^CCR7^−^), and TEMRA (CD45RA^+^CCR7^−^). **(H)** Spearman correlation between the frequencies of CD85j^+^ and TEMRA^+^ cells within CMV- (red) and EBV- (blue) specific CD8 T cell population. Frequencies of **(I)** CD85j^+^ and **(J)** TEMRA^+^ CMV-specific CD8 T cells in individuals with high (>1%) and low (<1%) tetramer^+^ cell frequencies. *p*-values were determined using Wilcoxon non-parametric paired **(B,D)** or Mann–Whitney unpaired **(I,J)** test.

CMV^+^ individuals expressed the highest frequencies of CD85j^+^ TEMRAs, thus we further investigated the expression of CD85j on CMV- and EBV-specific CD8 T cells from older CMV^+^ individuals. Directly *ex vivo*, both CMV (pp65) and EBV (BRLF1) tetramer-positive CD8 T cells were detectable (Figure 2A) but CMV-specific CD8 T cells were present at much higher frequencies than EBV-specific cells (median 1.2 vs. 0.29%, respectively) (Figure 2B). Expression of CD85j was detectable on CMV- and EBV-specific CD8 T cells, in particular on CD28^−^ effector cells (Figure 2C). Surprisingly, the expression of CD85j was similar on CMV- and EBV-specific cells (Figure 2D). Moreover, CD85j expression on CMV- and EBV-specific cells from the same individuals positively correlated (Figure 2E), suggesting that the probability of CD85j expression is not influenced by the nature of antigenic stimulation or the extent of clonal expansion, but by response patterns unique to individuals.

To further understand why the frequencies of CD85j^+^ cells varied between individuals, irrespective of whether specific for CMV or EBV, we characterized the phenotypic composition of CD85j^+^ CMV- and EBV-specific CD8 T cell populations. Expression of CD85j on CD8 T cell subsets was also confirmed in total CD8 T cells and CMV-specific CD8 T cells, demonstrating again robust expression on effector cells (Figure 2F). Both CMV- and EBV-specific populations of CD85j^+^ cells were predominated by effector populations (TEMRAs and EM cells), although CD85j^+^ CMV-specific cells had a higher median frequency of TEMRAs than EBV (Figure 2G). Similar to results from total CD8 T cells (Figure 1), the frequency of CD85j^+^ CMV- and EBV-specific cells positively also correlated with the frequency of TEMRA^+^ CMV- and EBV-specific cells (Figure 2H). However, individuals with high frequencies of CMV-specific CD8 T cells, which is indicative of memory inflation, exhibited increased percentage of cells expression CD85j and of the TEMRA subset (Figures 2I,J), suggesting that CD85j is gained during differentiation and retained during expansion of antigen-specific terminal-differentiated effector cells.

### TCR Repertoire of CD85j^+^ and CD85j^−^ CMV-Specific CD8 T Cells in Older Individuals

**Figure 3:**
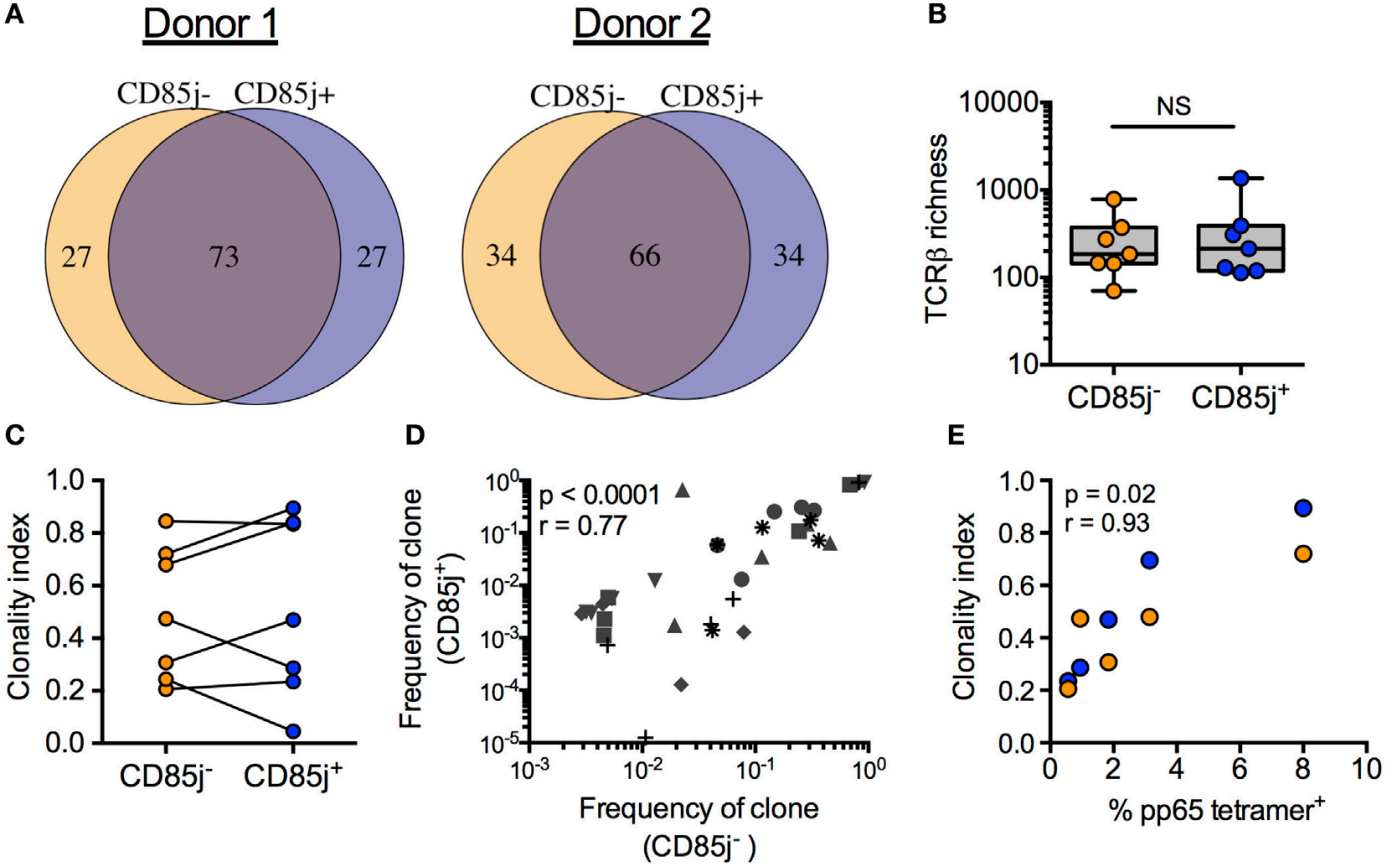
*TRB* sequence diversity in CD85j^+^ and CD85j^−^ cytomegalovirus (CMV) pp65-specific CD8 T cells. **(A)** Overlap of the top 100 most frequent *TRB* sequences from pp65-specific CD85j^+^ and CD85j^−^ CD8 T cell populations in two representative donors. **(B)** TCR richness (the number of unique *TRB* sequences) and **(C)** clonality index (a modified Gini Simpson index to estimate contribution of clonally expanded sequences within a repertoire) in pp65-specific CD85j^−^ and CD85j^+^ CD8 T cells from seven older individuals. **(D)** Spearman correlation between the frequencies of the top five clones in CD85j^−^ population with corresponding frequency in the CD85j^+^ CD8 T cells from seven older individuals, each of them represented by a different symbol. **(E)** Spearman correlation between the percentage of pp65-tetramer^+^ CD8 cells and *TRB* clonality index from CD85j^−^ (orange) and CD85j^+^ (blue) populations (*n* = 5). NS = not significant.

If CD85j expression is a late event in T effector cell expansion and differentiation, CD85j^+^ cells would be biased for larger clonal populations of antigen-specific T cells. Thus, we compared the TCR repertoires between CD85j^+^ and CD85j^−^ CMV-specific (pp65 tetramer-positive) CD8 T cells. The repertoires in individual donors largely overlapped indicating that both subsets derived from the same progenitor cells (Figure 3A). The total number of unique TRB sequences (TCR richness) was similar between the two populations, with median counts of 213 (range: 113–1,361) and 185 (range: 70–781) for CD85j^+^ and CD85j^−^ populations, respectively (Figure 3B). *TRB* sequences unique for one subset were all highly infrequent and, therefore, likely reflected the low probability of reidentification rather than uniqueness for one particular subset. Sizes of T cell clonotypes in both subsets showed a similar logarithmic distribution with a few clones dominant and no depletion of infrequent clones in either subset. Accordingly, clonality indices, a measure of clonal expansion, were not different (Figure 3C). In addition, there was a strong correlation between individual TCR clone frequencies in CD85j^+^ and CD85j^−^ populations (Figure 3D). Clonality indices also positively correlated with frequencies of CMV-specific CD8 T cells (Figure 3E). Thus, clonality reflects memory inflation but this clonal expansion occurs in the CD85j^+^ as well as CD85j^−^ subsets and CD85j expression does not appear to increase with clonal size or to have major influence on selection of the antigen-specific repertoire.

### Phenotypic and Functional Characterization of CD85j^+^ CMV-Responsive CD8 T Cells in Older Individuals

In initial studies using flow cytometry, stimulation with immediate early or late CMV antigens revealed that CD85j^+^ CD8 T cells produced significant levels of IFNγ (Figure S2 in Supplementary Material). To further interrogate the functional role of CD85j in antigen-specific responses, we utilized mass cytometry to determine simultaneous expression changes of 30 different parameters on CD8 T cells after CMV-specific peptide stimulation with immediate early or late antigen peptide super pools. These parameters included 5 T cell phenotyping markers, 7 cytokines, 2 cytotoxic factors, and 12 markers related to activation, exhaustion, and/or senescence. We termed all CD8 T cells expressing CD107a or one of the following cytokines, IFNγ, TNFα, IL-2, GM-CSF, and MIP1β, after stimulation as “CMV-responsive.”

**Figure 4:**
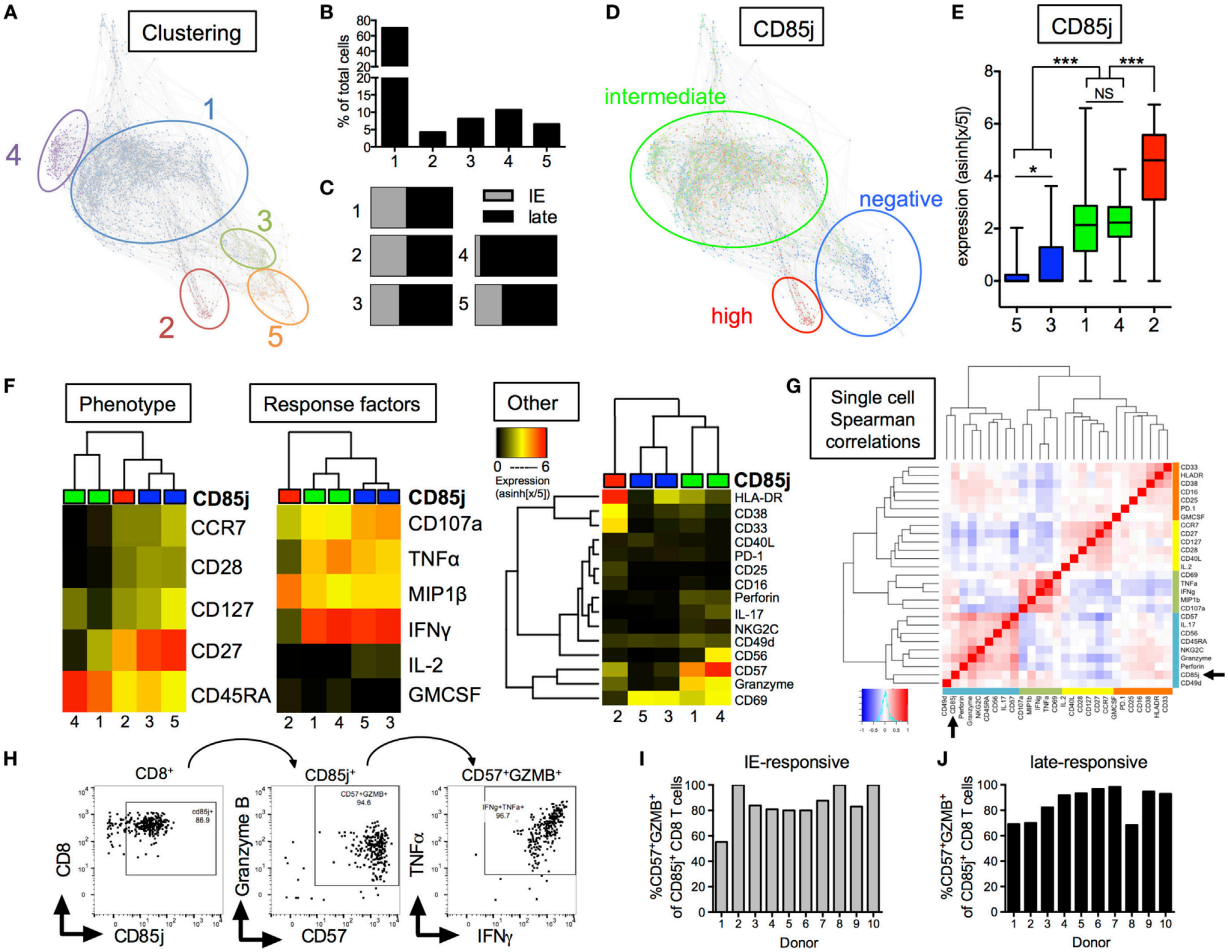
CyTOF analysis of CD85j expression on cytomegalovirus (CMV)-stimulated CD8 T cells from older individuals. **(A)** Single-cell force-directed layout of X-shift clustering (*K* = 55) of all CMV-responsive CD8 T cells from CMV^+^ elderly individual PBMCs (*n* = 30) stimulated for 18 h with peptide pools from immediate early (IE) and late CMV antigens, then stained for surface and intracellular markers. CMV-responsive CD8 T cells were defined as live/CD3^+^CD19^−^/CD8^+^CD4^−^ cells expressing IFNγ, TNFα, IL-2, GM-CSF, MIP1β, or CD107a after stimulation. Color code shows X-shift clusters. **(B)** Frequencies of cells with the individual clusters compared with all CMV-responsive cells. **(C)** Distribution of CMV-responsive CD8 T cells from IE or late peptide stimulation within each cluster. **(D)** Overlay of CD85j expression by individual cells within X-shift clusters. **(E)** CD85j expression on single cells from each of the X-shift clusters with *p*-values determined by one-way ANOVA. **(F)** Hierarchical clustering using phenotypic, CMV peptide stimulation-induced effector molecules, and other markers of activation, senescence, and exhaustion. Heat maps show median expression levels from each cluster, independent of CD85j expression. **(G)** Spearman correlation heat map of single-cell expression of all phenotypic, cytokine, and other markers from all CMV-responsive CD8 T cells. **(H)** Hand-gating of CD8^+^/CD85j^+^/CD57^+^GZMB^+^ cells in CMV-responsive cells from one representative donor after late peptide stimulation. Frequencies of CD57^+^GZMB^+^ cells in CD85j^+^ CD8 T cells from 10 individual donors **(I)** after IE or **(J)** late peptide stimulation. *p < 0.05, **p < 0.01, and ***p < 0.001.

From our global clustering analysis, we found five distinct clusters of CMV-responsive cells (Figure 4A). The most prominent cluster, cluster 1, included 70.3% of all CMV-responsive cells (Figure 4B) and displayed no significant difference in cell frequencies between the two different peptide stimulations (Figure 4C). Cluster 4 was the only cluster that showed differences based on peptide stimulation, where most cells within this cluster were found with late, but not immediate early, peptide stimulation. CD85j expression differentiated into three groups; CD85j-negative, -intermediate, and -high (Figure 4D). At a single-cell expression level, clusters 3 and 5 were CD85j-neg/low, clusters 1 and 4 were CD85j-intermediate, and cluster 2 was CD85j-high (Figure 4E). Overall, CD85j^intermediate^ and CD85j^−^ clusters made up 81.0 and 14.7% of the CMV-responsive population, respectively. The smallest fraction of CMV-responsive cells was CD85j^high^ (cluster 2: 4.3% of all cells). The analysis of functional markers shows that CD85j^high^ cells expressed high levels of MIP1β and CD33. Further analysis revealed that this MIP1β^+^CD33^+^CD85j^high^ population was already present in unstimulated cells (Figures S3A,B in Supplementary Material). Therefore, it is undetermined whether these CD85j^high^ cells are truly CMV-specific T cells or a population of myeloid cells.

Median expression of (1) phenotypic markers, (2) response cytokines, and (3) other markers were individually compared between the five clusters using unbiased hierarchal clustering (Figure 4F). CD85j^intermediate^ clusters separated from CD85j^−^ and CD85j^high^ clusters in all comparisons. CD85j^intermediate^ clusters (clusters 1 and 4) strongly separated from CD85j^−^ clusters by phenotypic but not stimulation-induced cytokine expression. Similar to previous analysis of CD85j^+^ cell phenotypes, we found that CD85j^intermediate^ clusters contained primarily terminally differentiated (CD45RA^+^CCR7^−^CD28^−^) CD8 T cells, whereas CD85j^−^ cells were CD27^+^ memory or naïve-like populations. Functionally, both CD85j^intermediate^ and CD85j^−^ cells expressed high levels of IFNγ, TNFα, and CD107a. However, the CD85j^intermediate^ population uniquely coexpressed high levels of CD57, granzyme B, and perforin, which are common markers of senescent cells or meditators of CTL responses (Figure 4F; Figure S4 in Supplementary Material). A subset of CD85j^intermediate^ cells from cluster 4 also coexpressed CD56 and NKG2C. Little to no expression of PD-1, an exhaustion marker, was detected on these cells. Median expression values and *p*-values for individual marker comparison between the different clusters are provided in Table S2 in Supplementary Material. Single-cell expression analysis of all CMV-responsive CD8 T cells demonstrated CD85j, CD57, and granzyme B expression positively correlated and clustered with NK or innate markers, CD56, perforin, and NKG2C (Figure 4G). In addition, hand-gating of CMV-responsive CD8 T cells confirmed that the majority of CD85j^+^ cells coexpress CD57 and granzyme B (Figure 4H), and coexpression is maintained across multiple donors and CMV antigen responses (Figures 4I,J). CD85j^+^CD57^+^granzymeB^+^ CD8 T cells were also found in the unstimulated population and single-cell analysis again revealed that CD57 and granzyme B positively correlated with CD85j in the resting CD8 T cell population (Figure S3 in Supplementary Material). Thus, CD85j^+^ CMV-responsive CD8 T cells are phenotypically and functionally similar to that of terminally differentiated, not exhausted, CD8 T cells.

### Inhibition of Proliferation but Not Cytokine Production by CD85j in CMV-Specific CD8 T Cells in Older Individuals

**Figure 5:**
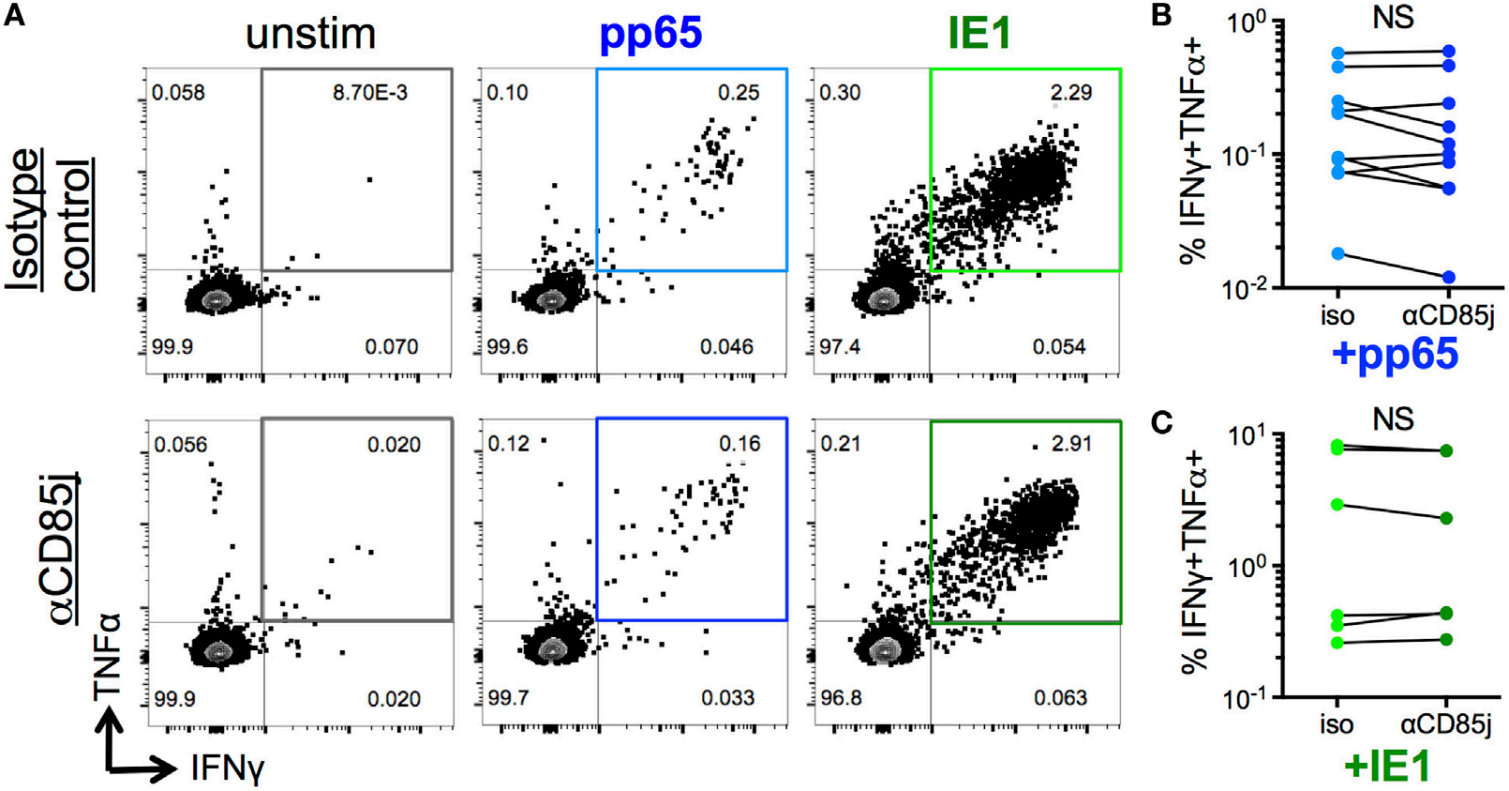
Function of CD85j in cytomegalovirus (CMV) peptide-induced cytokine responses in CD8 T cells. **(A)** Representative flow plots of IFNγ and TNFα expression by CD8 T cells after stimulation with pp65- or IE-1-peptide pools in the presence of anti-CD85j or isotype control antibody for 13 h. **(B,C)** Frequencies of IFNγ^+^TNFα^+^ CD8 T cells after stimulation with **(B)** pp65 (*n* = 10) and **(C)** IE-1 peptide pools (*n* = 7). *p*-values were determined using the Wilcoxon test. NS = not significant.

“Senescent” CD8 T cells are classically characterized by a reduction in proliferative capacity but maintained cytokine production, unlike exhausted cells, which also lost the ability to produce effector cytokines. Thus, we determined the potential role of CD85j in antigen-specific cytokine responses and proliferation in CMV-infected, older individuals. Consistent with the functional data from CyTOF analysis (Figure 4), stimulation of PBMCs with immediate early (IE-1) and late (pp65) CMV antigen peptide pools both induced simultaneous production of IFNγ and TNFα by a subset of CD8 T cells (Figure 5A). However, blocking CD85j did not affect the frequencies of CD8 cells producing IFNγ and TNFα upon pp65 and IE-1 stimulation (Figures 5B,C). Thus, CD85j does not appear to play a role in the functional ability of CD8 T cells to produce cytokines in response to antigen.

**Figure 6:**
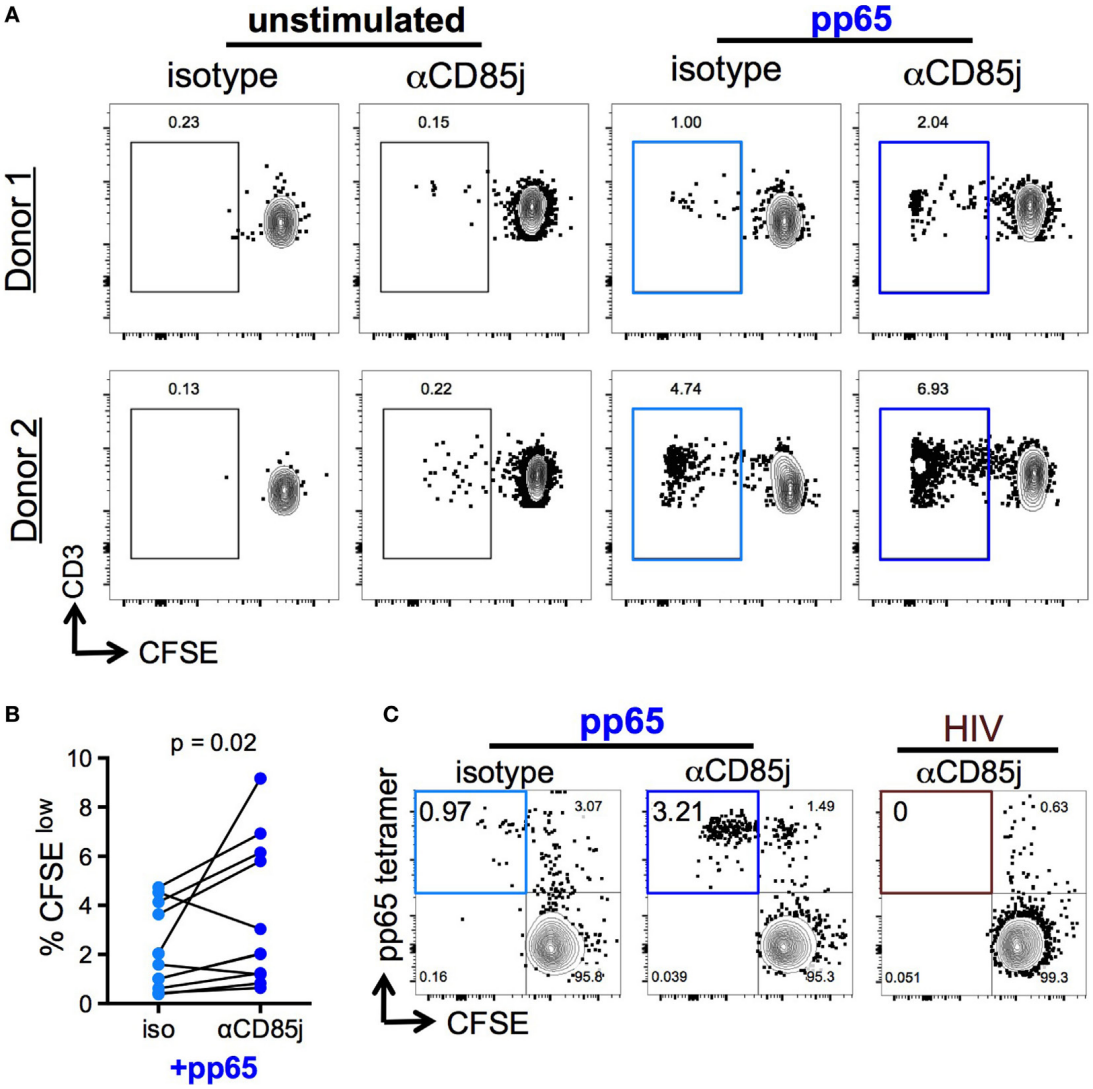
Function of CD85j in cytomegalovirus (CMV) peptide-induced proliferation in CD8 T cells. **(A,B)** CFSE-labeled PBMCs stimulated with or without pp65 peptide pool in the presence of anti-CD85j or isotype control antibody for 7 days. **(A)** Representative flow plots of CFSE staining in CD8 T cells (gated on live/CD3^+^/CD8^+^CD4^−^) from two different donors. **(B)** The frequency of CFSE^low^ (proliferated) CD8 T cells in stimulated PBMCs (*n* = 12) in the presence of anti-CD85j or isotype antibodies. **(C)** Flow plot of pp65-tetramer^+^ cell proliferation in total CD8 T cell isolated from CMV^+^ HLA-A2^+^ PBMCs and stimulated with pp65-loaded tetramer-coated plates for 7 days, in the presence of anti-CD85j or isotype control antibody. HIV peptide-loaded tetramers were used as a negative control. These data are representative of two experiments. *p*-values were determined using the Wilcoxon test.

Stimulation with pp65 peptide pool induced proliferation (CFSE^low^ cells) of a low-frequent T cell population (Figures 6A,B). Unlike cytokine production, CD85j blocking demonstrated a significant increase in the frequencies of proliferating CD8 T cells. CD85j blocking alone without stimulation was not sufficient to cause proliferation (Figure 6A; Figure S5A in Supplementary Material). To establish whether this inhibitory effect was specifically blocking CD85j engagement on CD8 T cells and not on CD85j-expressing antigen-presenting cells, we developed a cell culture system using immobilized pp65-loaded HLA-A*0201 tetramers to stimulate purified CD8 T cell *in vitro*. As observed with total PBMCs, CD85j blocking enhanced proliferation of CMV-specific CD8 T cells (Figure 6C; Figure S5B in Supplementary Material). Thus, engagement of CD85j on CD8 T cells reduced proliferation in response to antigen, indicating that CD85j may be a checkpoint regulator inhibiting clonal expansion of virus-specific T cells during aging.

## Discussion

Negative regulatory receptors on T cells, such as PD-1 and CTLA-4, are important checkpoint inhibitors that can be targeted to unleash T cell responses and improve the immunological control of tumors and, potentially, chronic viral infections. It is, therefore, of great interest whether this basic concept of checkpoint inhibition can also be applied to immune aging; where adaptive immunity and, in particular, CD8 T cell function falters and results in less efficient control of viral infections. Here, we focused on the inhibitory receptor CD85j and determined its cellular specificity and function in antigen-specific CD8 T cell responses during immune aging. We found that CD8 T cells acquire CD85j with effector cell differentiation, irrespective of the extent of clonal expansion, with maleness and age as predisposing factors. The association of CD85j expression with CMV infection is mainly due to terminal effector cell differentiation of CMV-specific CD8 T cells, not antigen-specificity. Moreover, the major function of CD85j is to constrain proliferation and clonal expansion, without impeding effector function upon antigenic stimulation. Together, these data indicate that CD85j plays an important function in checkpoint regulation during immune aging and that interfering with CD85j function may be useful to increase the frequencies of virus-specific T cells in older individuals.

Structurally, CD85j has high resemblance with the canonical checkpoint molecule PD-1, which has two ITIMs and one ITSM motif in its intracellular tail (29). Like PD-1, CD85j also demonstrates immune-suppressive functions in multiple cell types (30–32). These similarities suggest that CD85j may be a unique immune checkpoint factor and raises the possibility that CD85j plays an analogous role to that of PD-1 in anti-viral responses in immune aging. Indeed, previous studies using cross-linking of CD85j and CD3 have shown that CD85j can inhibit multiple cellular functions including cytokine production and cytotoxicity (33–35). The finding that a high frequency of CMV-specific T cells express CD85j permitted the phenotypic and functional comparison of antigen-specific CD85j^+^ and CD85j^−^ T cells. CyTOF cluster analysis after CMV peptide stimulation clearly showed that CD85j expression does not differentiate antigen-specific effector function of CD8 subsets. Phenotypically, CD85j^+^ cells most closely resemble TEMRAs, although a subset of EM can also express CD85j. However, these cells do not express PD-1, but more frequently the senescence marker CD57 and, therefore, are clearly different from exhausted cells. Indeed, these cells exhibit robust cytokine production and highest expression of granzyme B and perforin, demonstrating the retention of cytotoxic functionally.

It was surprising that we did not find any effect of CD85j on cytokine production after peptide stimulation, although the inhibitory function of CD85j is clearly directly related to MHC recognition; engagement of MHC-I by CD85j promotes intracellular inhibitory signaling and competitive binding of MHC-I with CD8 prevents positive costimulatory signaling (17). One possible explanation for our findings is that the inhibitory signals of CD85j are not of sufficient strength to block T cell activation. CD8 effector cells are also frequently of sufficient avidity to no longer require coreceptor engagement (36–38), so blocking CD8 engagement may not strongly influence antigen-induced cytokine production in TEMRAs. Moreover, the expression of CD85j on the surface of T cells is much lower than on other cell types on which it is expressed, due to lineage-specific differences in the transcriptional regulation (11). Additionally, although CD85j recognizes HLA-A and -B molecules, it has much higher affinity for HLA-G (17) and may therefore exhibit a lower inhibitory capacity in the context of our HLA-A *in vitro* stimulation system. However, we did see a distinct inhibitory activity of CD85j on the peptide-induced proliferative activity of CMV-specific cells that could be overcome by a blocking antibody. To confirm that the increased proliferative response was due to blocking CD85j on the responding T cells and not on the antigen-presenting cells, we used immobilized tetramers as peptide-presenting units in the absence of antigen-presenting cells and found essentially the same results. Therefore, it appears that proliferative responses by effector CD8 T cells are more sensitive to CD85j inhibitory signaling than cytokine production, consistent with our previous observation that sustained TCR signaling in TEMRAs, curtailed by engaging killer immunoglobulin-like receptors, leads to inhibition of proliferation but not cytotoxic activity (39). Mechanistically, it has been shown that partial phosphorylation of TCR-CD3 ITAMs reduces proliferation without affecting cytokine production (40), thus CD85j may functionally inhibit or reduce TCR-CD3 ITAM phosphorylation.

In aging humans, CD85j seems to dampen unopposed clonal expansion while leaving effector CD8 T cell functions intact. Such a mechanism could be very beneficial in the setting of chronic or latent viral infections such as by herpes viruses. Memory inflation is frequently seen with CMV infection to an extent that may compromise the overall T cell repertoire and therefore would have a negative impact on the ability to generate an immune response to unrelated antigens. Both memory inflation and frequencies of CD85j^+^ effector T cells show high interindividual variability. We did not see any evidence for an inverse correlation between CD85j expression and memory inflation that would have indicated that failure to express CD85j is a risk factor for unopposed expansion.

It is of interest to note that CD8 TEMRAs employ several mechanisms in addition to CD85j expression to the same effect, namely curtailing proliferation while maintaining effector function. Akbar and colleagues have recently identified a disproportionate activation of the p38 MAPK pathway in TEMRAs that is directly involved in the loss of telomerase activity and proliferative capacity as well as the increased production of inflammatory cytokines (41). Activation of p38 is a consequence of DNA damage responses involving ATM and mitochondrial dysfunction causing ROS production (42). The failure to proliferate associated with upregulated production of inflammatory cytokines is reminiscent of the senescence-associated secretory phenotype in fibroblasts, and similarly involves the cyclin-dependent kinase inhibitors p16 and p21 (43). However, unlike cellular senescence in a strict sense, these functional deficits are reversed by inhibiting p38 (44, 45). Therefore, two pathways, p38 activation as well as CD85j expression, appear to be important checkpoints employed by effector T cells to maintain T cell homeostasis while keeping control of latent viral infection during immune aging.

If CD85j’s function is to set a ceiling for clonal expansion in settings of chronic stimulation, one would expect that CD85j’ expression correlates with clonal size. Surprisingly, this was not the case. Expression of CD85j was similar for CMV and EBV peptide responses although the frequencies of cells specific for these viral peptides differed by an order of magnitude. Large interindividual variations in CD85j expression on antigen-specific T cells correlated better with differentiation into TEMRAs than clonal sizes. Interestingly, maleness as well as age were demographic variables that were associated with CD85j expression and TEMRA frequencies. Most convincingly, the TCR repertoire of CD85j^+^ and CD85j^−^ CMV pp65-specific CD8 T cells was very similar; in particular, the CD85j^+^ population was not enriched for large clones. These data suggest that CD85j expression occurs during effector cell differentiation irrespective of how many divisions a particular clone has undergone. They also show that CD85j does not induce a contraction of the antigen-specific repertoire, which is functionally important since it has been shown that not only the size of the CMV response but also the TCR diversity of CMV-specific cells determines how well CMV latency is retained (46).

Although CD85j may be an important checkpoint to maintain T cell homeostasis with age, it may also be harmful in certain contexts by excessively constraining clonal expansion. During aging, latent CMV infection is well-controlled without reactivation and there is no significant age-associated morbidity, while varicella zoster virus escapes latency causing shingles in up to 50% of individuals by the age of 80 years (47, 48). One possible reason for differential immune control is that the CMV response maintains the ability to undergo cellular expansion whereas VZV-specific T cells are unable to expand, causing a decline in the frequency of VZV-specific T cells with age (49, 50). Understanding the fine-tuning of signaling pathways involved in cellular proliferation, as well as effector functions, is a critical component to immune responsiveness. In the case of CMV infection, CD85j may provide a potential intervention to dampen excessive proliferation, while maintaining repertoire diversity and effector functions. Alternatively, inhibition of CD85j activity may be one way to improve anti-viral responses to other pathogens, such as VZV, with aging, similar to what has been proposed for the p38 pathway checkpoint. In addition to blocking CD85j’s ability to interact with its ligand, one alternative intervention is to target CD85j transcription. Such an approach would be particularly attractive, since CD85j is widely expressed on hematopoietic cells but transcriptional and translational control is lineage-specific (11) and would, therefore, allow a more selective targeting of CD85j in TEMRAs.

## Ethics Statement

This study was in accordance with the Declaration of Helsinki, approved by the Stanford Institutional Review Board, and all participants gave written informed consent.

## Author Contributions

CG: data collection, data analysis and interpretation, and manuscript writing. QQ: study design, data collection and analysis, and manuscript correction. JS and EN: study design and data collection. SG: data collection and analysis. RJ: data analysis and interpretation. HM: conception and design of study and financial support. CW: conception and design of study, data interpretation, and financial support. JG: conception and design of study, data analysis and interpretation, financial support, and manuscript writing. All the authors read, critiqued, and approved the final manuscript and also agreed to be accountable for all aspects of the work.

## Conflict of Interest Statement

The authors declare that the research was conducted in the absence of any commercial or financial relationships that could be construed as a potential conflict of interest.
