# Supplementary material for: Immune Checkpoint Function of CD85j in CD8 T Cell Differentiation and Aging
Source: Front Immunol. 2017 Jun 14;8:692. doi: 10.3389/fimmu.2017.00692 (PMC5469909; doi:10.3389/fimmu.2017.00692)
Supplement: Supplementary file 1 [file Data_Sheet_1.PDF]

Supplementary Material

Immune Checkpoint Function of CD85j in CD8 T Cell Differentiation and Aging

Claire E. Gustafson#, Qian Qi#, Jessica Hutter Saunders, Sheena Gupta, Rohit Jadhav, Evan Newell, Holden Maecker, Cornelia M. Weyand, Jörg J. Goronzy*

# co-first authors

*** Correspondence:** Jörg J. Goronzy, M.D., Ph.D.: jgoronzy@stanford.edu

1 Supplementary Figures

1.1 Supplemental Figure 1.

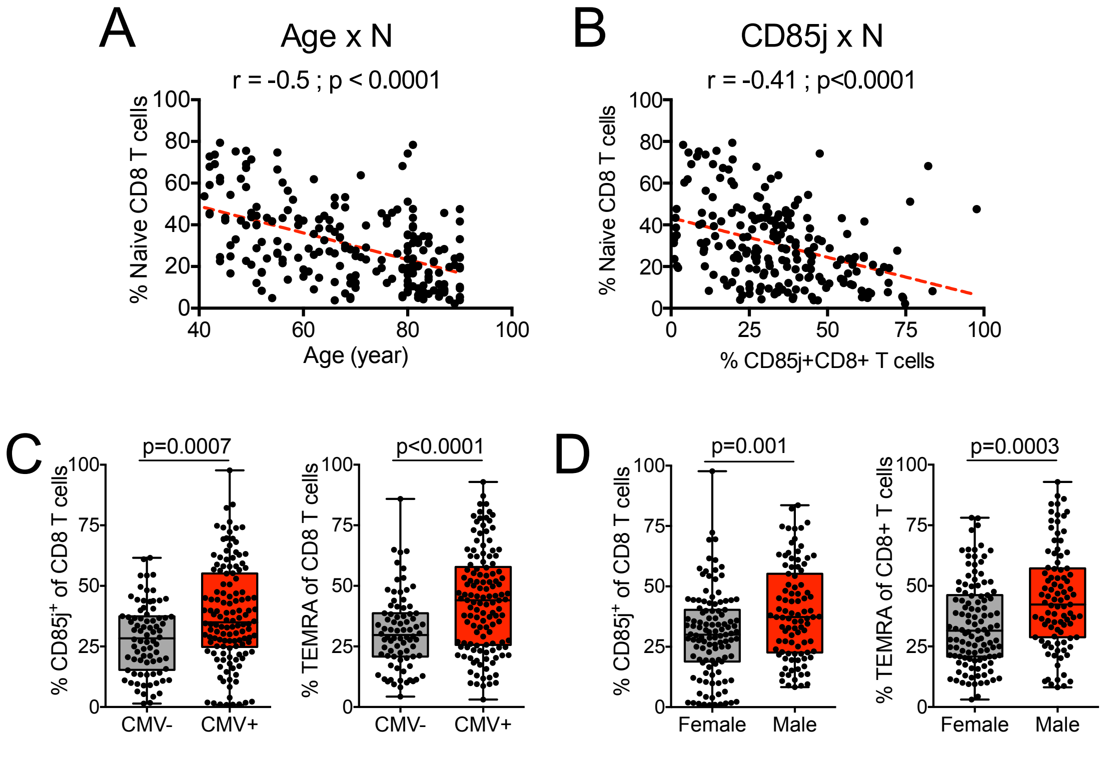

Supp Figure 1. Relationship between CD85j and T cell subsets, CMV status and gender. (A) Spearman correlation between age and the percent of naïve ($CD45RA^{+}CCR7^{+}$) CD8 T cells from 210 healthy individuals. (B) Spearman correlation between the percent of $CD85j^{+}$ cells and the percent of naïve ($CD45RA^{+}CCR7^{+}$) CD8 T cells. **(C-D)** The percent of $CD85j^{+}$ CD8 T cells and the percent of TEMRAs in total CD8 T cells separated by **(C)** CMV status and **(D)** gender.

1.2 Supplemental Figure 2.

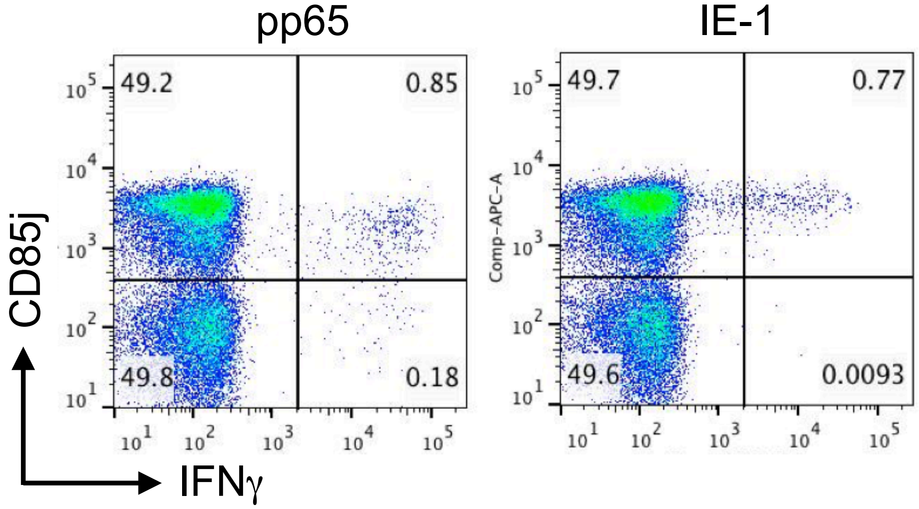

Supp Figure 2. IFNγ production by CD85j^{+} CD8 T cells after CMV peptide stimulation. PBMCs were stimulated with 1μg/ml CMV pp65 or IE-1 overlapping peptide pool for 13 hours in the presence of 10μg/ml brefeldin A. Cells were gated on CD8 T cells and analyzed for CD85j expression and IFNγ production.

1.3 Supplemental Figure 3.

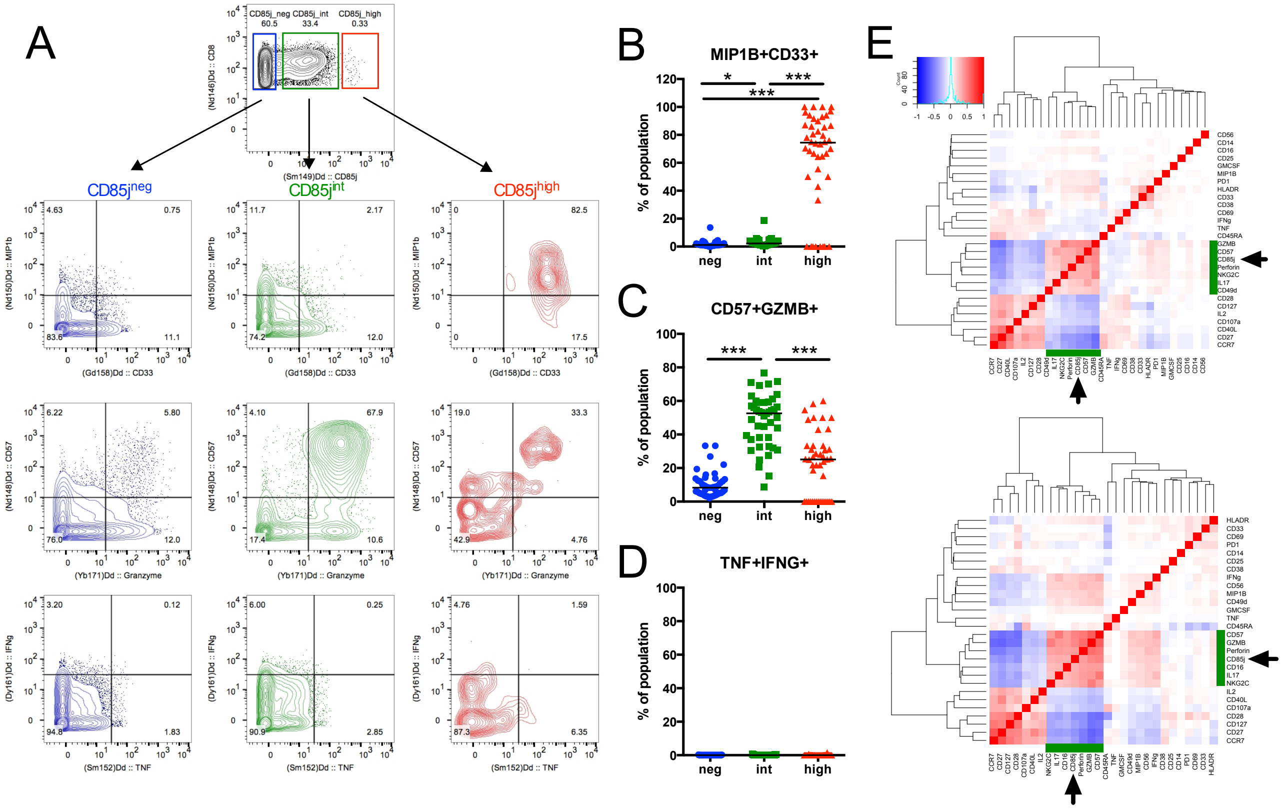

Supp Figure 3. Ex vivo phenotyping of CD85j populations without prior stimulation. (A) Representative flow plots of CD33 and MIP1β (top panels), GZMB and CD57 (middle panels), TNFα and IFNγ (bottom panels) expression on CD85j-negative (blue), CD85j-intermediate (green) and CD85j-high (red) populations in CD8 T cells from unstimulated PBMCs samples. **(B-D)** Frequencies of **(B)** MIP1β^{+}CD33^{+}, **(C)** CD57^{+}GZMB^{+} and **(D)** TNFα^{+}IFNγ^{+} populations from individual unstimulated PBMC samples (n=44). **(E)** Representative Spearman correlations of single cell (CD8^{+} T cell) expression of individual markers from unstimulated PBMC from two donors. Arrows highlight CD85j. Green bars indicates closest hierarchical cluster containing CD85j.

1.4 Supplemental Figure 4.

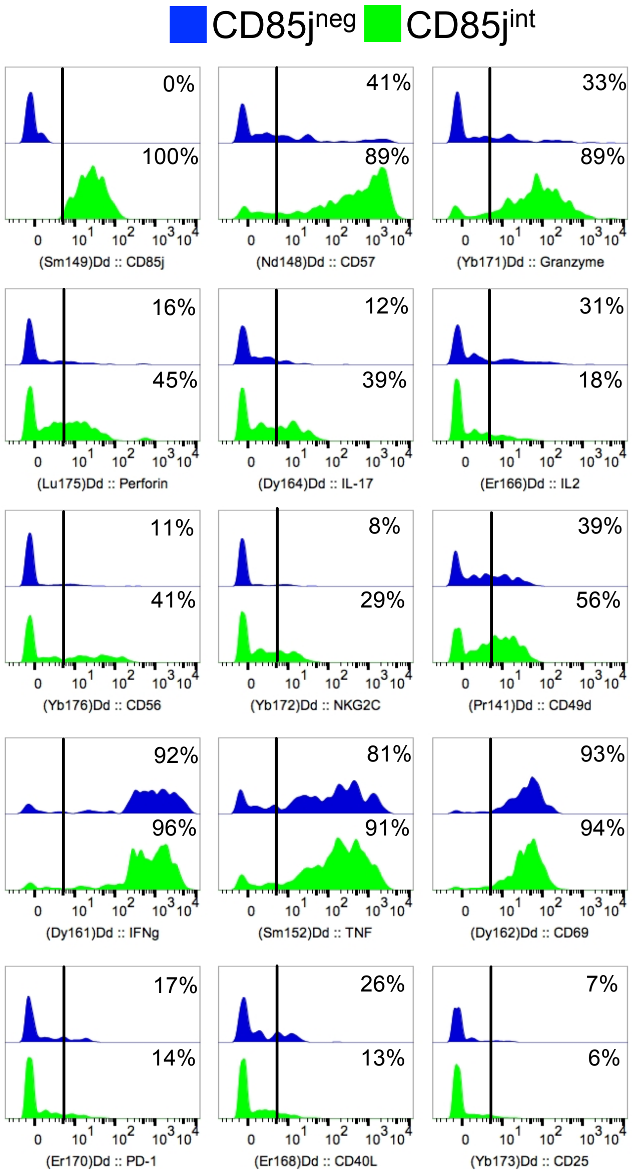

Supp Figure 4. Expression of surface markers and cytokines by CD85j-negative and CD85j-intermediate (int) CD8 T cells. CMV-responsive $CD8^+$ T cells from pp65 peptide pool-stimulated PBMCs were hand-gated for CD85j expression. CMV-responsive CD8 T cells were defined as Live/$CD3^+CD19^-$/$CD8^+CD4^-$ cells expressing IFNγ, TNFα, IL-2, GM-CSF, MIP1β or CD107a after stimulation. Expression of markers on CD85j-negative (blue) and CD85j-intermediate (green) cells is shown for a representative CyTOF staining. Gates were consistently set at 5 for all markers to exclude most false positives, as this is a conservative upper bound of background in any CyTOF channels.

1.5 Supplemental Figure 5.

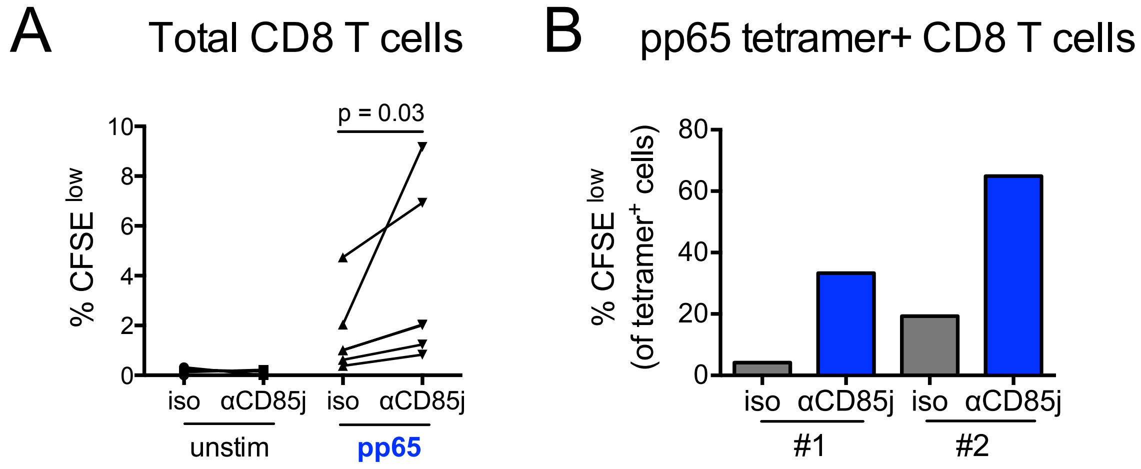

Supp Figure 5. CD85j and proliferation of CD8 T cells. (A) Frequencies of proliferated ($CFSE^{low}$) CD8 T cells cultured in medium only (unstim) or stimulated with pp65 peptide pool in the presence or absence of CD85j blocking antibody or isotype control for 7 days. P-values were determined using Wilcoxon test. **(B)** Percent of proliferated ($CFSE^{low}$) pp65-tetramer^{+} CD8 T cells within all pp65-tetramer^{+} CD8 T cells from two individual CMV^{+} HLA-A2^{+} donors stimulated with pp65-loaded tetramer-coated plates for 7 days, in the presence of anti-CD85j or isotype control antibody.

2 Supplementary Tables

2.1 Supplemental Table 1. CyTOF antibody panel

Base panel	Atomic Mass
cell length	N/A
DNA	Ir191, Ir193
Live/Dead	In115
CD3	Sm154
CD19	Nd142
CD14	Gd160
CD4	Nd145
CD8	Nd146
TCRgd	Dy163
**Phenotypic markers**	**Atomic Mass**
CD45RA	Eu153
CCR7	Tm169
CD28	Gd155
CD27	Er167
CD127	Ho165
**Response Cyotkines**	**Atomic Mass**
IFNg	Dy161
TNFa	Sm152
IL-2	Er166
GM-CSF	Tb159
MIP1b	Nd150
CD107a	Eu151
**Other markers**	**Atomic Mass**
CD85j	Sm149
Granzyme B	Yb171
perforin	Lu175
IL-17	Dy164
HLA-DR	Gd157
CD38	Gd156
CD69	Dy162
CD40L	Er168
PD-1	Er170
CD25	Yb173
CD16	Yb174
CD33	Gd158
CD56	Yb176
CD57	Nd148
CD49d	Pr141
NKG2C	Yb172

2.2 Supplemental Table 2.

A

	Average expression of cluster					p-value (Kruskal-Wallis with Dunn's multiple comparison test)										
Phenotypic markers	1	2	3	4	5	K-W	1v2	1v3	1v4	1v5	2v3	2v4	2v5	3v4	3v5	4v5
CCR7	0.32	0.85	0.85	0.15	1.16	< 0.0001	< 0.0001	< 0.0001	ns	< 0.0001	ns	< 0.0001	ns	< 0.0001	ns	< 0.0001
CD28	0.25	0.79	0.97	0.21	0.95	< 0.0001	< 0.0001	< 0.0001	ns	< 0.0001	ns	< 0.0001	ns	< 0.0001	ns	< 0.0001
CD127	0.38	0.94	1.07	0.77	1.42	< 0.0001	< 0.0001	< 0.0001	< 0.0001	< 0.0001	ns	ns	< 0.001	< 0.05	< 0.05	< 0.0001
CD27	1.08	1.96	2.50	0.34	2.63	< 0.0001	< 0.0001	< 0.0001	< 0.0001	< 0.0001	ns	< 0.0001	< 0.05	< 0.0001	ns	< 0.0001
CD45RA	2.31	1.62	1.79	2.85	1.45	< 0.0001	< 0.0001	< 0.0001	< 0.0001	< 0.0001	ns	< 0.0001	ns	< 0.0001	ns	< 0.0001

B

	Average expression of cluster					p-value (Kruskal-Wallis with Dunn's multiple comparison test)										
Response factors	1	2	3	4	5	K-W	1v2	1v3	1v4	1v5	2v3	2v4	2v5	3v4	3v5	4v5
CD107a	3.14	2.35	4.06	2.86	3.87	< 0.0001	< 0.01	< 0.0001	ns	< 0.001	< 0.0001	ns	< 0.0001	< 0.0001	ns	< 0.0001
TNFa	3.60	1.23	3.40	4.23	3.66	< 0.0001	< 0.0001	ns	< 0.001	ns	< 0.0001	< 0.0001	< 0.0001	< 0.001	ns	ns
MIP1B	3.45	4.38	3.29	2.90	3.30	< 0.0001	< 0.0001	ns	< 0.0001	ns	< 0.0001	< 0.0001	< 0.0001	< 0.01	ns	< 0.01
IFNg	4.94	1.21	5.62	5.45	5.07	< 0.0001	< 0.0001	< 0.0001	ns	ns	< 0.0001	< 0.0001	< 0.0001	ns	ns	ns
IL-2	0.35	0.41	0.91	0.32	0.93	0.0004	ns	< 0.0001	ns	< 0.0001	ns	ns	ns	< 0.001	ns	< 0.001
GMCSF	0.46	0.65	0.35	0.67	0.33	0.0033	ns	ns	< 0.001	ns	ns	ns	ns	< 0.01	ns	< 0.05

C

	Average expression of cluster					p-value (Kruskal-Wallis with Dunn's multiple comparison test)										
Other factors	1	2	3	4	5	K-W	1v2	1v3	1v4	1v5	2v3	2v4	2v5	3v4	3v5	4v5
HLA-DR	1.84	5.39	2.44	0.92	0.36	< 0.0001	< 0.0001	< 0.0001	< 0.0001	< 0.0001	< 0.0001	< 0.0001	< 0.0001	< 0.0001	< 0.0001	< 0.001
CD38	0.87	2.93	0.67	0.36	0.28	< 0.0001	< 0.0001	ns	< 0.0001	< 0.0001	< 0.0001	< 0.0001	< 0.0001	< 0.001	< 0.0001	ns
CD33	0.51	3.24	0.35	0.23	0.20	< 0.0001	< 0.0001	ns	< 0.0001	< 0.001	< 0.0001	< 0.0001	< 0.0001	ns	ns	ns
CD40L	0.26	0.38	0.35	0.24	0.65	< 0.0001	ns	ns	ns	< 0.001	ns	< 0.05	ns	< 0.01	ns	< 0.0001
PD-1	0.42	0.39	0.38	0.25	0.30	0.048	ns	ns	ns	ns	ns	ns	ns	ns	ns	ns
CD25	0.15	0.78	0.17	0.13	0.15	< 0.0001	< 0.0001	ns	ns	ns	< 0.0001	< 0.0001	< 0.0001	ns	ns	ns
CD16	0.31	0.53	0.09	0.17	0.07	< 0.0001	< 0.0001	ns	ns	< 0.001	< 0.0001	< 0.01	< 0.0001	ns	ns	< 0.01
Perforin	0.96	0.20	0.21	0.85	0.23	< 0.0001	< 0.0001	< 0.0001	ns	< 0.0001	ns	< 0.0001	ns	< 0.0001	ns	< 0.0001
IL-17	0.57	0.13	0.18	1.07	0.13	< 0.0001	< 0.0001	< 0.0001	< 0.0001	< 0.0001	ns	< 0.0001	ns	< 0.0001	ns	< 0.0001
NKG2C	0.53	0.28	0.03	0.37	0.05	< 0.0001	< 0.01	< 0.0001	ns	< 0.0001	< 0.01	ns	ns	< 0.0001	ns	< 0.0001
CD49d	1.15	0.65	0.72	0.96	0.76	< 0.0001	< 0.0001	< 0.0001	ns	< 0.0001	ns	ns	ns	ns	ns	ns
CD56	0.39	0.30	0.14	3.02	0.03	< 0.0001	ns	< 0.0001	< 0.0001	< 0.0001	< 0.05	< 0.0001	< 0.01	< 0.0001	ns	< 0.0001
CD57	4.19	1.89	0.60	5.74	0.25	< 0.0001	< 0.0001	< 0.0001	< 0.0001	< 0.0001	< 0.01	< 0.0001	< 0.0001	< 0.0001	ns	< 0.0001
Granzyme	3.37	1.17	0.32	2.81	0.23	< 0.0001	< 0.0001	< 0.0001	< 0.0001	< 0.0001	< 0.05	< 0.0001	< 0.01	< 0.0001	ns	< 0.0001
CD69	2.37	0.59	2.78	2.84	2.84	< 0.0001	< 0.0001	< 0.0001	< 0.0001	< 0.0001	< 0.0001	< 0.0001	< 0.0001	ns	ns	ns
CD85j	1.99	4.16	0.66	2.15	0.20	< 0.0001	< 0.0001	< 0.0001	ns	< 0.0001	< 0.0001	< 0.0001	< 0.0001	< 0.0001	< 0.05	< 0.0001

Supp Table 2. Comparison of (A) Phenotypic markers, (B) response cytokines and (C) other markers with CMV-responsive CD8 T cell clusters. Clusters are color-coded based on CD85j expression (see Figure 4D-F); blue is CD85j-negative, green is CD85j-intermediate and red is CD85j-high. The average expression of clusters are given as transformed expression values (asinh[x/5]). Red numbers indicate the cluster with highest expression and blue numbers indicates the cluster with the lowest expression. P-values for each marker were determined using Kruskal-Wallis test with Dunn's multiple comparison test.
